# Nostalgia increases punitiveness by intensifying moral concern

**DOI:** 10.1038/s41598-024-61858-x

**Published:** 2024-05-19

**Authors:** Jannine D. Lasaleta, Tim Wildschut, Constantine Sedikides

**Affiliations:** 1 Marketing Department, Sy Syms School of Business, Yeshiva University, New York, NY USA; 2 Center for Research on Self and Identity, School of Psychology, University of Southampton, Southampton, UK

**Keywords:** Psychology, Human behaviour

## Abstract

We addressed the relation between nostalgia and moral judgment or behavior. We hypothesized that nostalgia, a social emotion, increases moral concern (H1), nostalgia intensifies punitiveness against moral transgressors (H2), and that the nostalgia—punitiveness link is mediated by moral concern (H3). We conducted three cross-sectional (Studies 1, 2, 4) and one experimental (Study 3) investigations (*N* = 1145). The investigations, involving distinct operationalizations of the relevant constructs (nostalgia, moral concern, punitiveness) and diverse samples (U.S., Canadian, and European Prolific workers, French business school students, Dutch community members), yielded results consistent with the hypotheses. Nostalgia keeps one’s moral compass in check. The findings enrich the emotions and morality literatures.

## Introduction

Questions of morality pervade everyday life. Whether it be masking and vaccinations, gun control and mass shootings, abortion and access to healthcare, or systemic racial injustice, individuals frequently judge moral issues and respond to moral violations. What factors are associated with, or influence, such judgments and responses? Emotions constitute one such factor. The literature has focused on such discrete emotions as disgust^1–6^, anger^4–6^, gratitude^7,8^, as well as shame, guilt, and embarrassment^9,10^. No research, however, has addressed another discrete emotion, nostalgia. We do so in this article.

### Nostalgia and sociality

According to *The New Oxford Dictionary of English*, nostalgia, “a sentimental longing or wistful affection for the past” (p. 1266^11^), is experienced frequently (e.g., three to four times a week;^12,13^) by individuals across cultures^14,15^ and age groups^16,17^. The emotion is bittersweet: predominantly positive (“warm feeling about the past, a past that is imbued with happy memories, pleasures, and joy”^18^), but with tinges of sadness or longing^19,20^.

Extensive evidence indicates that nostalgia begets sociality^21–23^. Nostalgia is a self-referential and social emotion that entails reminders of others. For instance, the content of experimentally induced nostalgic narratives itself is social: these narratives comprise reflections of meaningful events (e.g., birthdays, graduations, family holidays) in which the self is encircled by close others (e.g., friends, partners, relatives)^13^. Trait nostalgia, defined as the proclivity to experience, and ascribe importance to, nostalgia^24^, is related to reminders of others as well^25^. Specifically, it positively predicts both relational collectivism, where the self-concept is embedded with close interpersonal relationships, and group collectivism, where the self-concept is embedded within a group^26^. Thus, in nostalgic reflection, “the mind is peopled”^27^, as one re-establishes a symbolic connection with influential others who are brought to life and blend in the present^28^. Nostalgia’s sociality is also demonstrated by its beneficial role in maintaining social relationships. For instance, trait nostalgia is positively associated with intimacy maintenance^29^. Further, experimentally induced nostalgia buffers the negative consequences of procedural injustice in organizations, strengthening cooperative attitudes^30^. Taken together, both trait and experimentally induced nostalgia are associated with or evoke thoughts of others, which conduce to decision-making and judgments that encourage group cohesiveness.

### Nostalgia and morality

We propose that nostalgia and morality are related. Nostalgia fosters sociality, which refers to social connections and interpersonal-relationship regulation. Individuals use moral values as guides to moral decisions that facilitate interpersonal functioning. We put forward the hypothesis that nostalgia increases moral concern, defined as the importance one assigns to being a good (vs. bad) person. Further, we hypothesize that the nostalgia-evoked increase in moral concern increases punitiveness, defined as the importance or severity of punishment meted out to a moral transgressor.

Moral values regulate social relationships by setting standards of how individuals should treat one another for the greater societal good^31,32^. Such standards include, for example, cooperation and group cohesiveness^33–35^. Here, we examine morality within Moral Foundations Theory (MFT)^31,32^, for the following reasons. First, MFT is a prominent, well-established theory, that has proven influential not only in psychology but also in other disciplines such as politics^36^, consumer research^37^, and music^38^. Second, MFT explains morality across cultures^39^, a focus of our research. Third, although other theories of morality are concerned with identity^40^ or development^41^, MFT provides a comprehensive framework that encompasses the spectrum of social-moral concerns.

Moral Foundations Theory stipulates that moral decision making and judgments lie within five moral foundations: Care/Harm, Fairness/Cheating, Loyalty/Betrayal, Authority/Subversion, and Sanctity/Degradation^31^. Care/Harm focuses on suffering avoidance, protection, kindness, and compassion toward others. Fairness/Cheating highlights reciprocity, proportionality, and altruism. Authority/Subversion is concerned with upholding hierarchical structures and traditions. Sanctity/Degradation relates to preventing physical, social, and spiritual contamination. Finally, Loyalty/Betrayal refers to commitments and obligations to the group. These five foundations can be further classified into two overarching categories, binding foundations (Loyalty/Betrayal, Authority/Subversion, Sanctity/Degradation), which emphasize regulating communal behavior, and individualizing foundations (Care/Harm, Fairness/Cheating), which emphasize the rights and welfare of individuals. We note that this framework has been criticized for the modularity and innateness of moral foundations^42^, the multiplicity as opposed to singularity of moral mechanisms^43^, and the generalizability of the findings across diverse populations (e.g., African Americans^44^). Evidence suggests, however, that the framework and the 5-factor structure of the relevant scale (Moral Foundations Questionnaire^31,32^) are a reasonable fit across diverse populations^45–47^.

The literature hints at the connection between nostalgia and moral values. First, the mere presence of others, even if imagined, amplifies moral values^48^. Nostalgia is related to reminders of meaningful interactions with significant others^13^; therefore, the nostalgia-associated reminders of others may increase moral concern. Second, nostalgia predicts collectivism^25^, which encompasses both intragroup and intergroup relationships. The emotion prescribes guidelines for individuals to make decisions that benefit interpersonal relationships^49^ or the group^14^; hence, nostalgia may be associated with moral concern. Third, nostalgia promotes prosocial attitudes and behaviors, such as cooperation^30,50^, charitable donations^51,52^, and prejudice reduction^53–56^, all of which can be perceived as sociomoral concerns linked to moral values^57^. Finally, nostalgia is linked to empathy^51^, which is implicated in moral decisions^58^. In summary, evidence points to nostalgia being a moral emotion, given that it involves others, and encourages sociomoral attitudes and behaviors^59–61^.

Crucially, this evidence maps onto the moral foundations. Nostalgia increases empathy and helping^51,52^; this corresponds to the Care/Harm foundation, which reflects suffering avoidance, protection, kindness, and compassion toward others. Therefore, nostalgia will be associated with, or intensify, sensitivity to violations of this moral foundation. Also, nostalgia is positively related to prejudice reduction and opposition to perceived injustice^53,62^; this corresponds to the Fairness/Cheating foundation, which reflects interest in fairness, reciprocal altruism, and co-operation. Therefore, nostalgia will be associated with, or intensify, sensitivity to unfairness. Moreover, nostalgia strengthens social ties and social stability^63–65^ as well as employees’ commitment to their organization and support for authorities^30,50,66^; this corresponds to the Loyalty/Betrayal and Authority/Subversion foundations, which reflect deference to leaders and traditions, as well as allegiance to one’s ingroup. Therefore, nostalgia will be associated with, or intensify, sensitivity to violations of these moral foundations. Finally, nostalgia imbues life with meaning^67,68^ and fosters intergenerational transfer of cultural traditions^69^; this entails a degree of correspondence to the Sanctity/Degradation foundation, which reflects the idea that some things, including life and venerated objects, should be treated with reverence and respect. These foundations encompass the dimensions of morality, all contributing to the goal of social survival. Nostalgia enhances sociality, placing emphasis on socio-moral concerns across these foundations. Put otherwise, nostalgia acts as a rising tide that lifts all moral boats.

We further hypothesize a downstream consequence of the nostalgia–moral concern link: increased punitiveness toward moral violations. Moral concern and punitiveness are positively related: Moral concern predicts harsh moral judgments and punishment intentions toward those who commit moral violations^70–73^. For example, endorsement of each moral foundation predicts stronger attitudes toward both utilitarian punishment (aimed at avoiding repetition of violations) and retributive punishment (aimed at retribution of violators). Accordingly, nostalgia, by virtue of its link to moral concern, will be associated with, or intensify, punitiveness toward those who inflict harm on others or shirk their duty of care (Care/Harm), behave unfairly or do not reciprocate accordingly (Fairness/Cheating), betray their group (Loyalty/Betrayals), create disorder or disobey authorities (Authority/Respect), and are toxic toward or contaminate the social system (Sanctity/Degradation).

The proposed link between nostalgia (a predominantly positive emotion) and greater punitiveness may seem counterintuitive in light of evidence that positive emotions often engender weaker moral judgment^57^. However, we advocate in favor of the fundamental sociality of nostalgia^21,22,74^ rather than its affective signature. Nostalgia entails opposition to, and disapproval of, behaviors that threaten social relationships and social stability. It is likely, then, that nostalgia, given its approach- or future-oriented property^75,76^, inspires people to stand up and defend their morals. If punishment is the only avenue afforded to them, they will choose it.

### Hypotheses and overview

We formulated three hypotheses. First, nostalgia positively predicts and increases moral concern (H1). Second, nostalgia positively predicts and increases punitiveness toward moral transgressors (H2). Third, the link between nostalgia and punitiveness is mediated by moral concern (H3). Given that nostalgia promotes sociality and that all foundations reflect socio-moral concerns, we theorize that nostalgia has a global influence on both moral concern and punitiveness.

We tested these hypotheses in four studies. In cross-sectional Study 1, we examined whether dispositional nostalgia predicts increased moral concern and ensuing punitiveness toward moral transgressors. In preregistered Study 2, we examined the replicability of Study 1. In experimental Study 3, we examined whether experimentally-induced nostalgia increases moral concern and resultant punitiveness toward moral transgressors. In cross-sectional Study 4, we tested the generalizability of Studies 1–3 in relation to justice sensitivity (to operationalize moral concern) and penal attitudes (to operationalize punitiveness) using a nationally representative sample.

In accord with our hypotheses, we tested the global influence of nostalgia on moral concern and punitiveness, rather than the differential influence of nostalgia on each foundation-specific moral concern and foundation-specific punitiveness. Although moral foundations are often treated separately, they also work together, as a system, to support social survival. Therefore, we focused on overall moral concern and punitiveness in Studies 1–3.

### Transparency and openness

All studies were approved by the ethics committee of the first author’s institution, WCG IRB, and were conducted in accordance with the institutional guidelines and regulations. We obtained informed consent from all participants. We report the determination of our sample size, all manipulations, all measures, and all data exclusions, and we follow Journal Article Reporting Standards^77^. We preregistered Study 2 (https://aspredicted.org/blind.php?x=N5B_XYJ).

## Study 1

In Study 1, we examined the relations among nostalgia, moral concern, and punishment. Specifically, we tested the hypotheses that nostalgia positively predicts moral concern (H1) and punitiveness toward moral transgressors (H2). Further, we tested the hypothesis that moral concern mediates the association between nostalgia and punishment (H3). We focused on the links among trait nostalgia and moral concern across the five moral foundations, and on punitiveness with regard to moral violations in these five domains.

### Method

#### Participants

We aimed for a sample size equal to or greater than 250 participants, to obtain stable initial estimates of the associations among trait nostalgia, moral concern, and punitiveness^78^. We exceeded this target and recruited 399 Prolific workers for $1.55. We removed four participants who reported being less than 18 years of age, that is, younger than the Prolific age requirement. Inclusion of these four participants’ responses did not affect the results. The final sample comprised 395 participants (206 women, 189 men), aged between 18 and 71 years (*M* = 32.86, *SD* = 11.36). They were native English speakers from the U.S. (*n* = 177), Canada (*n* = 26), U.K. (*n* = 183), and Republic of Ireland (*n* = 7; two participants did not report their nationality). We conducted a post-hoc power analysis with the MedPower application^79^ to determine achieved power for detecting the indirect effect of nostalgia on punitiveness via moral concern. Achieved power approximated 100%.

#### Materials and procedure

We assessed nostalgia and moral concern in counterbalanced order. Assessment of punitiveness followed.

**Nostalgia.** We assessed this construct with the Southampton Nostalgia Scale (SNS^15,61^; α = 0.94, *M* = 4.40, *SD* = 1.33). This 7-item scale includes the definition of nostalgia (‘a sentimental longing for the past’) followed by items measuring perceived importance (e.g., “How valuable is nostalgia for you?”; 1 = *not at all*, 7 = *very much*) and frequency (e.g., “How often do you experience nostalgia?”; 1 = *not at all*, 7 = *very much*) of nostalgic engagement.

**Moral Concern.** We assessed this construct using the Moral Foundations Questionnaire (MFQ^78^). The MFQ contains 30 items that measure five moral foundations (six items per foundation): (1) Care/Harm, (2) Fairness/Cheating, (3) Loyalty/Betrayal, (4) Authority/Subversion, and (5) Sanctity/Degradation. The MFQ comprised two parts. In Part I, participants judged the moral relevance of 15 scenarios (e.g., “Whether or not someone cared for someone weak or vulnerable” [for Care/Harm]; 0 = *not at all relevant*, 5 = *extremely relevant*). In Part II, participants rated their level of agreement with 15 moral judgment statements (e.g., “Justice is the most important requirement for a society” [for Fairness/Cheating]; 0 = *strongly disagree*, 5 = *strongly agree*). Taken together, these scores reflect the degree to which participants prioritize moral concerns; that is, higher scores indicate the importance one places on being a good (vs. bad) person. We aggregated scores across the two parts, creating five foundation-specific indices of moral concern: Care/Harm (α = 0.65, *M* = 3.72, *SD* = 0.69); Fairness/Reciprocity (α = 0.71, *M* = 3.64, *SD* = 0.70); Loyalty/Betrayal (α = 0.71, *M* = 2.39, *SD* = 0.83); Authority/Subversion (α = 0.73, *M* = 2.59, *SD* = 0.87); and Sanctity/Degradation (α = 0.80, *M* = 2.18, *SD* = 1.06).

**Punitiveness.** We assessed this construct by instructing participants to rate their attitude toward punishment for 10 moral-violation scenarios related to the five moral foundations (i.e., two scenarios per moral foundation, based on Study 3 from Graham and colleagues^80^). For each moral foundation, participants read two scenarios, one describing a slight (e.g., “Dana stepped on ant hill, killing thousands of ants”) and another a moderate (e.g., “Chris made cruel remarks to an overweight person about his or her appearance”) foundation-related violation. For each scenario, we assessed participants’ attitude toward punishing the depicted moral violation, by averaging the following two items: “How important is to punish [name] for this action?” (1 = *not at all*, 7 = *very important*) and “How severely, if at all, should [name] be punished?” (1 = *not at all*, 7 = *very severely*). We intended for these measures to reflect the degree to which participants regarded it important to penalize someone for committing a moral transgression across various moral domains, and how severe that penalty ought to be. Higher scores indicate greater punitiveness. We randomized the order of the 10 scenarios. We created five foundation-specific punitiveness indices by averaging the scores on the four items (2 scenarios × 2 ratings), given that we had not formulated different hypotheses for level of moral violation severity: Care/Harm (α = 0.80, *M* = 3.12, *SD* = 1.13); Fairness/Reciprocity (α = 0.79, *M* = 3.08, *SD* = 1.11); Loyalty/Betrayal (α = 0.88, *M* = 1.28, *SD* = 0.70); Authority/Subversion (α = 0.89, *M* = 3.62, *SD* = 1.35); and Sanctity/Degradation (α = 0.79, *M* = 2.68, *SD* = 1.43).

### Results

We note that, when reporting *F* tests, we present the 90% CI for eta-squared because the *F* distribution is one sided^81^. This ensures that inferences based on *p*-values will agree with the lower confidence limit. We present the 95% CI for correlation coefficients. When reporting mediation analyses, we present the 95% CI for standardized regression coefficients and 95% bootstrap confidence interval for standardized indirect effects.

#### Trait nostalgia and moral concern

We entered the five MFQ subscales in a mixed Analysis of Covariance (ANCOVA), with trait nostalgia as a between-subjects covariate and moral foundation (Care/Harm vs. Fairness/Cheating vs. Loyalty/Betrayal vs. Authority/Subversion vs. Sanctity/Degradation) as a within-subjects variable. Across the five moral foundations, trait nostalgia predicted higher moral concern (i.e., a main effect of trait nostalgia on the average moral concern rating across the five moral foundations), *F*(1, 393) = 33.40, *p* < 0.001, η^2^ = 0.078, 90% CI [0.041, 0.123]. The Trait Nostalgia × Moral Foundation interaction was not significant, indicating that the association between trait nostalgia and moral concern did not vary as a function of foundation, *F*(4, 1572) = 0.15, *p* = 0.964, η^2^ = 0.000, 90% CI [0.000, 0.001]. Tests of simple associations revealed that trait nostalgia was significantly correlated with elevated moral concern within each of the five foundations (Table 1). These findings support H1.

**Table 1:** Correlations between trait nostalgia and moral concern in Study 1 (*N* = 399).

	Nostalgia	Care/Harm	Fairness/Cheating	Loyalty/Betrayal	Authority/Subversion	Sanctity/Degradation	Overall moral concern
Nostalgia	–	[0.129, 0.317]	[0.092, 0.282]	[0.106, 0.296]	[0.088, 0.279]	[0.039, 0.233]	[0.186, 0.368]
Care/Harm	0.225***	–	[0.540, 0.665]	[− 0.092, 0.105]	[− 0.055, 0.142]	[− 0.026, 0.170]	[0.373, 0.530]
Fairness/Cheating	0.188***	0.607***	–	[− 0.129, 0.068]	[− 0.176, 0.020]	[− 0.159, 0.037]	[0.264, 0.436]
Loyalty/Betrayal	0.203***	0.006	− 0.031	–	[0.620, 0.727]	[0.532, 0.659]	[0.700, 0.787]
Authority/Subversion	0.185***	0.044	− 0.079	0.677***	–	[0.668, 0.764]	[0.756, 0.829]
Sanctity/Degradation	0.137**	0.073	− 0.061	0.599***	0.719***	–	[0.766, 0.836]
Overall moral concern	0.280***	0.455***	0.353***	0.747***	0.795***	0.804***	–

Numbers below diagonal are correlation coefficients. Numbers above diagonal are 95% CI for correlation coefficients.

** *p* < .01. *** *p* < .001.

#### Trait nostalgia and punitiveness

We entered the punitiveness ratings in a mixed ANCOVA, with trait nostalgia as a between-subjects covariate, and moral foundation (Care/Harm vs. Fairness/Cheating vs. Loyalty/Betrayal vs. Authority/Subversion vs. Sanctity/Degradation) as a within-subjects variable. Across the five moral foundations, trait nostalgia predicted higher punitiveness (i.e., a main effect of trait nostalgia on the average punitiveness rating across the five moral foundations), *F*(1, 393) = 4.17, *p* = 0.042, η^2^ = 0.010, 90% CI [0.0002, 0.033]. The Trait Nostalgia × Moral Foundation interaction was not significant, indicating that the association between nostalgia and punitiveness did not vary as a function of foundation, *F*(4, 1572) = 0.80, *p* = 0.525, η^2^ = 0.002, 90% CI [0.000, 0.005]. Trait nostalgia was positively correlated with elevated punitiveness within all five foundations, but the correlation was trending for the Authority/Subversion and Sanctity/Degradation foundations (Table 2). These results are generally consistent with H2.

**Table 2:** Correlations between trait nostalgia and punitiveness in Study 1 (*N* = 399).

	Nostalgia	Care/Harm	Fairness/Cheating	Loyalty/Betrayal	Authority/Subversion	Sanctity/Degradation	Overall punitiveness
Nostalgia	–	[− 0.043, 0.153]	[− 0.024, 0.172]	[− 0.068, 0.129]	[− 0.006, 0.190]	[− 0.015, 0.181]	[0.004, 0.199]
Care/Harm	0.056	–	[0.296, 0.465]	[0.167, 0.351]	[0.314, 0.480]	[0.237, 0.413]	[0.625, 0.731]
Fairness/Cheating	0.075	0.384***	–	[0.207, 0.387]	[0.395, 0.549]	[0.224, 0.402]	[0.653, 0.753]
Loyalty/Betrayal	0.031	0.262***	0.299***	–	[0.125, 0.313]	[0.313, 0.479]	[0.471, 0.610]
Authority/Subversion	0.093^+^	0.400***	0.476***	0.221***	–	[0.340, 0.502]	[0.722, 0.804]
Sanctity/Degradation	0.084^+^	0.328***	0.316***	0.399***	0.424***	–	[0.697, 0.785]
Overall punitiveness	0.102*	0.682***	0.707***	0.544***	0.766***	0.744***	–

Numbers below diagonal are correlation coefficients. Numbers above diagonal are 95% CI for correlation coefficients.

^+^ *p* < .10. * *p* < .05. *** *p* < .001.

#### Mediation analyses

The positive associations of trait nostalgia with, respectively, moral concern and punitiveness were not qualified by moral foundation. For the purpose of mediation analyses, we therefore created overall measures of moral concern and punitiveness by averaging the pertinent scores across moral foundations*.* Trait nostalgia was positively associated with these composite measures of moral concern, *r*(395) = 0.280, *p* < 0.001, 95% CI [0.186, 0.368], and punitiveness, *r*(395) = 0.102, *p* = 0.042, 95% CI [0.004, 0.199]. (These correlations are mathematically equivalent to the main effects of nostalgia in the above-reported mixed ANCOVAs.) Additionally, the moral-concern composite was positively associated with the punitiveness composite, *r*(395) = 0.382, *p* < 0.001, 95% CI [0.295, 0.464].

We tested our hypothesis that moral concern mediates the link between nostalgia and punitiveness using Hayes’s PROCESS macro^82^ (Model 4; 10,000 bootstrap samples). We report standardized parameter estimates (b*). When controlling for trait nostalgia, the association between moral concern and punitiveness was significant (path *b*), b* = 0.384, *t*(392) = 7.90, *p* < 0.001, 95% CI [0.288, 0.479]. When controlling for moral concern, the direct effect of trait nostalgia on punitiveness was not significant (path *c’*), b* = − 0.005, *t*(392) = − 0.10, *p* = 0.919, 95% CI [− 0.101, 0.091]. Consistent with H3, there was a significant indirect effect (path *ab*) of nostalgia on punitiveness via moral concern, b* = 0.107, 95% CI = [0.059, 0.162].

#### Model comparison

We used SAS Proc Calis to compare the hypothesized model (nostalgia ⇒ moral concern ⇒ punitiveness) to an alternative model, in which the order of moral concern and punitiveness was reversed (nostalgia ⇒ punitiveness ⇒ moral concern). To do so, we tested two full-mediation models where the entire effect of the predictor on the outcome is transmitted via the mediator. These models do not include a residual direct effect of the predictor on the outcome and are therefore non-saturated. Inclusion of the direct effect would result in a saturated model and perfect model fit. Furthermore, inclusion of the direct effect would result in two models with the same paths between the same variables. Any two models that have the same paths between the same variables will have the same fit, even if some paths are in a different direction^83^.

We report two measures of absolute fit: the Χ^2^ test (with the understanding that it is sensitive to sample size) and standardized root mean square residual (SRMR). We do not report the root mean square error of approximation (RMSEA), because models with few degrees of freedom, such as ours, can have artificially large RMSEA values^84^. An SRMR value smaller than 0.08 is considered indicative of good fit^85^. Given our interest in comparing competing models, we further report two parsimony fit indices: the Akaike Information Criterion (AIC)^86^ and Bayesian Information Criterion^87^. Within a set of models for the same data, AIC and BIC can be used to compare competing models that need not be nested (smaller values are better).

The hypothesized model had excellent fit, Χ^2^(1) = 0.01, *p* = 0.918, SRMR = 0.002, AIC = 10.01, BIC = 29.90. The alternative model had poor fit, Χ^2^(1) = 27.99, *p* < 0.001, SRMR = 0.098, AIC = 37.99, BIC = 57.89. The model comparison favored the hypothesized model, in which moral concern precedes punitiveness.

### Discussion

Averaged across moral foundations, trait nostalgia predicted stronger moral concern (H1) and higher punitiveness (H2). Additionally, the relation between trait nostalgia and punitiveness was mediated by moral concern (H3). The omnibus analysis of punitiveness scores revealed a significant overall effect of nostalgia that was not qualified by moral foundation. We acknowledge, however, that the numerically small correlations of nostalgia with punitiveness per foundation (Table 2) may raise concerns regarding the replicability of Study 1 findings. To address this issue, we replicated and extended Study 1 in preregistered Study 2.

## Study 2

Study 2 was a preregistered replication of Study 1. To bolster the construct validity of trait nostalgia, we assessed it with three convergent scales^88,89^.

### Method

#### Participants

Using Study 1 results as input, sample size calculations with the MedPower application^79^ stipulated a minimum sample size of 102 to detect an indirect effect of nostalgia on punitiveness via moral concern (80% power, two-tailed α = 0.05). To obtain stable estimates, we exceeded this and aimed for a minimum sample size of *N* = 250, as in Study 1^78^. We recruited 276 Prolific workers, remunerating them with $3.20, and excluded 18 for failing an attention check. We paid participants a higher amount than in Study 1, because Study 2 was longer, and acceptable payment rates along with service fees increased between 2016 (when we completed Study 1) and 2022 (when we completed Study 2). The final sample comprised 258 participants (150 men, 107 women, 1 undisclosed), aged between 19 and 78 years (*M* = 45.07, *SD* = 14.04). They were native English speakers from the U.K. (*n* = 212), U.S. (*n* = 27), and Canada (*n* = 15). There was one participant each from Nigeria, Philippines, and Hong Kong, and one participant who did not report their nationality.

#### Materials and procedure

We first assessed trait nostalgia with three scales, presented in a separate random order, to validate our operationalization of the construct. (For a similar practice, see Kelley et al.^90^) The first one was the SNS^61^ (α = 0.96, *M* = 4.40, *SD* = 1.44). The second one was the Personal Inventory of Nostalgic Experiences (PINE^91^; α = 0.93, *M* = 4.36, *SD* = 1.53). It contains four items that measure the extent to which people long for the past (e.g., “How much do you feel a wistful affection for the past?’; 1 = *not at all*, 7 = *very much*). The third one was the Nostalgia Prototype Scale (NPS^53^; α = 0.92, *M* = 4.33, *SD* = 1.24). Participants are presented with five statements based on prototypical features of nostalgia (e.g., “I bring to mind rose-tinted memories”^12^) and then indicate how often they engage in these activities (1 = *I do this rarely,* 7 = *I do this a lot*) and how important it is for them to do so (1 = *this is not important to me,* 7 = *this is very important to me*), producing a total of 10 ratings (5 statements × 2 ratings).

Next, participants completed the same measures of moral concern and punitiveness as in Study 1. We created five foundation-specific indices of moral concern: Care/Harm (α = 0.73, *M* = 3.70, *SD* = 0.74); Fairness/Reciprocity (α = 0.69, *M* = 3.67, *SD* = 0.70); Loyalty/Betrayal (α = 0.74, *M* = 2.20, *SD* = 0.89); Authority/Subversion (α = 0.76, *M* = 2.42, *SD* = 0.89); and Sanctity/Degradation (α = 0.81, *M* = 2.19, *SD* = 1.04). We also created five foundation-specific punitiveness indices: Care/Harm (α = 0.83, *M* = 3.10, *SD* = 1.19); Fairness/Reciprocity (α = 0.78, *M* = 3.20, *SD* = 1.09); Loyalty/Betrayal (α = 0.84, *M* = 1.18, *SD* = 0.49); Authority/Subversion (α = 0.86, *M* = 3.33, *SD* = 1.21); Sanctity/Degradation (α = 0.78, *M* = 2.63, *SD* = 1.31). As in Study 1, we randomized the order of the punitiveness scenarios. Embedded in these measures was our attention check (“I am currently attending a university that does not exist”; 0 = *strongly disagree*, 5 = *strongly agree*). We excluded participants who reported scores greater than 0 (*n* = 18). As preregistered, we also assessed empathy with an 8-item version of Mehrabian and Epstein’s scale^92^ for exploratory purposes. We only analyzed and report the measures that test our key hypotheses.

### Results

#### Nostalgia measures composite

The three nostalgia scales were highly correlated (*r*s > 0.81, *p*s < 0.001), replicating findings of Kelley et al.^90^. Therefore, we standardized and then averaged the scales to create a composite nostalgia index. We used this index in all subsequent analyses. We note that we tested a single-factor model for the three nostalgia scales, using confirmatory factor analysis. To identify the model, we constrained the item error variances to be equal. Model fit was excellent, Χ^2^(2) = 2.27, *p* = 0.322, SRMR = 0.001, CFI = 1.00 (see also Wildschut et al.^24^). We obtained similar results for each scale (Supplementary Information, Tables S1–S3).

#### Trait nostalgia and moral concern

We entered the five MFQ subscales in a mixed ANCOVA, with trait nostalgia as a between-subjects covariate and moral foundation (Care/Harm vs. Fairness/Cheating vs. Loyalty/Betrayal vs. Authority/Subversion vs. Sanctity/Degradation) as a within-subjects variable. Across the five moral foundations, trait nostalgia predicted higher moral concern (i.e., a main effect of trait nostalgia on the average moral concern rating across the five moral foundations), *F*(1, 256) = 32.83, *p* < 0.001, η^2^ = 0.114, 90% CI [0.059, 0.176]. Unlike in Study 1, the Trait Nostalgia × Moral Foundation interaction was significant, *F*(4, 1024) = 5.83, *p* < 0.001, η^2^ = 0.022, 90% CI [0.007, 0.036], indicating that the association between trait nostalgia and moral concern did vary as a function of foundation. This interaction was ordinal. Tests of simple associations revealed that trait nostalgia was significantly positively related with four of the five moral concern foundations (all *p*s < 0.01, except for the Fairness/Cheating dimension: *r* = 0.07, *p* = 0.261; Table 3). These results are consistent with H1.

**Table 3:** Correlations between trait nostalgia and moral concern in Study 2 (*N* = 258).

	Nostalgia	Care/Harm	Fairness/Cheating	Loyalty/Betrayal	Authority/Subversion	Sanctity/Degradation	Overall moral concern
Nostalgia	–	[0.041, 0.279]	[− 0.052, 0.191]	[0.202, 0.422]	[0.135, 0.364]	[0.169, 0.394]	[0.224, 0.441]
Care/Harm	0.163**	–	[0.592, 0.729]	[0.031, 0.270]	[− 0.121, 0.123]	[0.069, 0.304]	[0.440, 0.615]
Fairness/Cheating	0.070	0.666***	–	[− 0.119, 0.126]	[− 0.221, 0.021]	[− 0.091, 0.153]	[0.286, 0.493]
Loyalty/Betrayal	0.316***	0.153*	0.004	–	[0.620, 0.749]	[0.532, 0.685]	[0.730, 0.826]
Authority/Subversion	0.253***	.001	− 0.102	0.690***	–	[0.625, 0.753]	[0.689, 0.797]
Sanctity/Degradation	0.286***	0.189**	0.032	0.615***	0.695***	–	[0.776, 0.857]
Overall moral concern	0.337***	0.533***	0.395***	0.783***	0.748***	0.820***	–

Numbers below diagonal are correlation coefficients. Numbers above diagonal are 95% CI for correlation coefficients.

* *p* < .05. ** *p* < .01. *** *p* < .001.

#### Trait nostalgia and punitiveness

We entered the punitiveness ratings in a mixed ANCOVA, with trait nostalgia as a between-subjects covariate and moral foundation (Care/Harm vs. Fairness/Cheating vs. Loyalty/Betrayal vs. Authority/Subversion vs. Sanctity/Degradation) as a within-subjects variable. Across the five moral foundations, trait nostalgia predicted higher punitiveness (i.e., a main effect of trait nostalgia on the average punitiveness rating across the five moral foundations), *F*(1, 256) = 6.17, *p* = 0.014, η^2^ = 0.024, 90% CI [0.003, 0.062]. The Trait Nostalgia × Moral Foundation interaction was not significant, *F*(4, 1024) = 1.96, *p* = 0.099, η^2^ = 0.008, 90% CI [0.000, 0.015], indicating that the relation between nostalgia and punitiveness did not vary significantly across foundations. Trait nostalgia was positively correlated with elevated punitiveness within all five foundations, but the correlation was significant for the Loyalty/Betrayal and Sanctity/Degradation foundations only (Table 4). However, the absence of a significant Trait Nostalgia × Moral Foundation interaction lends confidence to the hypothesis that nostalgia is overall related to higher punitiveness, regardless of moral foundation.

**Table 4:** Correlations between trait nostalgia and punitiveness in Study 2 (*N* = 258).

	Nostalgia	Care/Harm	Fairness/Cheating	Loyalty/Betrayal	Authority/Subversion	Sanctity/Degradation	Overall punitiveness
Nostalgia	–	[− 0.040, 0.203]	[− 0.095, 0.149]	[0.051, 0.289]	[− 0.051, 0.192]	[0.068, 0.304]	[0.032, 0.270]
Care/Harm	0.083	–	[0.276, 0.485]	[0.091, 0.325]	[0.262, 0.473]	[0.202, 0.422]	[0.661, 0.778]
Fairness/Cheating	0.027	0.385***	–	[0.001, 0.241]	[0.331, 0.529]	[0.067, 0.303]	[0.594, 0.730]
Loyalty/Betrayal	0.172**	0.211***	0.123*	–	[0.144, 0.372]	[0.130, 0.360]	[0.321, 0.522]
Authority/Subversion	0.071	0.372***	0.435***	0.262***	–	[0.173, 0.397]	[0.678, 0.790]
Sanctity/Degradation	0.189**	0.316***	0.187**	0.248***	0.289***	–	[0.588, 0.726]
Overall punitiveness	0.153*	0.725***	0.668***	0.427***	0.739***	0.663***	–

Numbers below diagonal are correlation coefficients. Numbers above diagonal are 95% CI for correlation coefficients.

* *p* < .05. ** *p* < .01. *** *p* < .001.

#### Mediation analyses

The positive association of trait nostalgia with punitiveness was not qualified by moral foundation. The positive association of trait nostalgia with moral concern was qualified by moral foundation, but the interaction effect was ordinal (i.e., the relation of trait nostalgia with punitiveness was positive for each foundation). For the purpose of mediation analyses, we therefore created overall measures of moral concern and punitiveness by averaging the pertinent scores across foundations, as in Study 1*.* Trait nostalgia was positively associated with these composite measures of moral concern, *r*(258) = 0.337, *p* < 0.001, 95% CI [0.224, 0.441] and punitiveness, *r*(258) = 0.153, *p* = 0.014, 95% CI [0.032, 0.270]. Additionally, the moral-concern composite was positively associated with the punitiveness composite, *r*(258) = 0.420, *p* < 0.001, 95% CI [0.314, 0.516].

We tested our hypothesis that moral concern mediates the link between nostalgia and punitiveness using Hayes’s PROCESS macro^82^ (Model 4; 10,000 bootstrap samples). When controlling for trait nostalgia, the association between moral concern and punitiveness was significant (path *b*), b* = 0.416, *t*(255) = 6.89, *p* < 0.001, 95% CI [0.297, 0.535]. When controlling for moral concern, the direct effect of trait nostalgia on punitiveness was not significant (path *c’*), b* = 0.013, *t*(255) = 0.22, *p* = 0.826, 95% CI [-0.106, 0.132]. Consistent with H3, there was a significant indirect effect (path *ab*) of nostalgia on punitiveness via moral concern, b* = 0.140, 95% CI = [0.078, 0.218].

#### Model comparison

We compared the hypothesized model (nostalgia ⇒ moral concern ⇒ punitiveness) to an alternative model in which the order of moral concern and punitiveness was reversed (nostalgia ⇒ punitiveness ⇒ moral concern), as in Study 1. The hypothesized model had excellent fit, Χ^2^(1) = 0.05, *p* = 0.825, SRMR = 0.005, AIC = 10.05, BIC = 27.81. The alternative model had poor fit, Χ^2^(1) = 24.94, *p* < 0.001, SRMR = 0.111, AIC = 34.94, BIC = 52.70. The model comparison favored the hypothesized model.

### Discussion

In Study 2, we replicated Study 1’s findings, supporting H1-H3. The association between nostalgia and punitiveness was numerically small, as in Study 1, but replicable. Studies 1–2 compared two full-mediation models. In the hypothesized model (H3), moral concern preceded punitiveness, whereas this order was reversed in the alternative model. Results of both studies clearly supported the hypothesized model. But what about the position of nostalgia in the postulated causal chain? Our hypotheses propose that nostalgia precedes both moral concern (H1) and punitiveness (H2), but cross-sectional Studies 1–2 did not establish causal precedence. We addressed this lacuna in experimental Study 3.

## Study 3

In Study 3, we manipulated nostalgia and tested its causal effects on moral concern and punitiveness. We hypothesized that moral concern (H1) and punitiveness (H2) would be higher in the nostalgia than control condition, and that moral concern would mediate the effect of nostalgia (vs. control) on punitiveness (H3).

### Method

#### Participants

We aimed to achieve at least 80% power to detect the indirect effect of nostalgia on punitiveness via moral concern. Using Studies 1–2 results as input, sample size calculations with the MedPower application^79^ stipulated a minimum sample size between 101 (based on Study 1) and 72 (based on Study 2). We conservatively adopted the higher target and exceeded it. We tested 151 students at an international French Business School (87 women, 64 men), aged between 19 and 29 years (*M* = 21.63, *SD* = 1.58), who completed the study for course credit. Participants were enrolled in an English-based program and were screened using a self-reported English fluency score of at least 8 on an 11-point scale (1 = *not fluent at all,* 11 = *extremely fluent*; *M* = 8.82, *SD* = 0.91). They were from France (*n* = 128), China (*n* = 14), and Morocco (*n* = 6). There was one participant each from Algeria and Russia, and one participant who did not report their nationality. We randomly assigned participants to the nostalgia (*n* = 72) or control (*n* = 79) condition.

#### Materials and procedure

Participants first completed the Event Reflection Task^13,61^. In the nostalgia condition, they received the dictionary definition of nostalgia (“sentimental longing for the past”), reflected on a nostalgic event from their lives and how it made them felt, listed five keywords capturing the gist of the event, and described it in writing for 3.5 min. In the control condition, participants followed the same protocol but for an ordinary event from their lives. Participants were given the choice of writing in English or French.

Next, participants completed a 3-item manipulation check (e.g., “I feel nostalgic at the moment”^12,13^; α = 0.96, *M* = 4.16, *SD* = 2.00) and the same measures of moral concern and punitiveness as in Study 1. We created five foundation-specific indices of moral concern: Care/Harm (α = 0.68, *M* = 2.67, *SD* = 1.07); Fairness/ Reciprocity (α = 0.75, *M* = 2.60, *SD* = 1.11); Loyalty/Betrayal (α = 0.62, *M* = 2.46, *SD* = 0.96); Authority/Subversion (α = 0.58, *M* = 2.30, *SD* = 0.89); and Sanctity/Degradation (α = 0.67, *M* = 1.91, *SD* = 0.96). We also created five foundation-specific punitiveness indices: Care/Harm (α = 0.66, *M* = 4.04, *SD* = 1.13); Fairness/Reciprocity (α = 0.81, *M* = 3.79, *SD* = 1.33); Loyalty/Betrayal (α = 0.83, *M* = 1.98, *SD* = 1.07); Authority/Subversion (α = 0.86, *M* = 5.02, *SD* = 1.28); and Sanctity/Degradation (α = 0.82, *M* = 3.49, *SD* = 1.52).

### Results

#### Manipulation check

As intended, participants in the nostalgia condition (*M* = 5.09, *SD* = 1.66) reported feeling more nostalgic than those in the control condition (*M* = 3.31, *SD* = 1.92), *F*(1, 149) = 36.64, *p* < 0.001, η^2^ = 0.197, 90% CI [0.109, 0.287]. The manipulation was effective.

#### Nostalgia and moral concern

We entered the five MFQ subscales in a mixed ANOVA, with nostalgia as a between-subjects variable and moral foundation (Care/Harm vs. Fairness/Cheating vs. Loyalty/Betrayal vs. Authority/Subversion vs. Sanctity/Degradation) as a within-subjects variable. Across the five moral foundations, nostalgia (compared to control) increased moral concern (i.e., a main effect of nostalgia on the average moral concern rating across the five moral foundations), *F*(1, 149) = 7.49, *p* = 0.007, η^2^ = 0.048, 90% CI = [0.008, 0.114]. The Nostalgia × Moral Foundation interaction was not significant, indicating that the effect of nostalgia (vs. control) on moral concern did not vary as a function of foundation, *F*(4, 596) = 0.85, *p* = 0.491, η^2^ = 0.006, 90% CI = [0.000, 0.013]. These findings are consistent with H1. We present tests of simple nostalgia effects within each moral foundation in Table 5.

**Table 5:** Means and standard deviations (in parentheses) for moral concern as a function of the nostalgia manipulation in Study 3.

Moral foundation	Control condition	Nostalgia condition	*F* (1, 149)	*p*	η^2^	95% CI
Care/harm	2.42 (1.21)	2.95 (0.82)	9.60	0.002	0.061	[0.013, 0.131]
Fairness/cheating	2.43 (1.24)	2.78 (0.93)	3.64	0.059	0.024	[0.000, 0.077]
Loyalty/betrayal	2.29 (1.05)	2.64 (0.82)	5.08	0.026	0.033	[0.002, 0.092]
Authority/subversion	2.14 (0.97)	2.49 (0.75)	6.10	0.015	0.039	[0.004, 0.101]
Sanctity/degradation	1.77 (1.03)	2.06 (0.86)	3.53	0.062	0.023	[0.000, 0.076]
Overall moral concern	2.21 (0.96)	2.58 (0.67)	7.49	0.007	0.048	[0.008, 0.114]

#### Nostalgia and punitiveness

We entered the punitiveness ratings in a mixed ANOVA, with nostalgia as a between-subjects covariate and moral foundation as a within-subjects variable. Across the five moral foundations, nostalgia (vs. control) increased overall punitiveness (i.e., a main effect of nostalgia on the average punitiveness rating across the five moral foundations), *F*(1, 149) = 4.35, *p* = 0.039, η^2^ = 0.028, 90% CI [0.001, 0.085]. The Nostalgia × Moral Foundation interaction was not significant, *F*(4, 596) = 1.00, *p* = 0.408, η^2^ = 0.007, 90% CI [0.000, 0.015]. The effect of nostalgia (vs. control) on punitiveness did not vary as a function of foundation. These findings support H2. We display tests of simple nostalgia effects within each moral foundation in Table 6.

**Table 6:** Means and standard deviations (in parentheses) for punitiveness as a function of the nostalgia manipulation in Study 3.

Moral foundation	Control condition	Nostalgia condition	*F* (1, 149)	*p*	η^2^	95% CI
Care/harm	3.97 (1.12)	4.13 (1.15)	0.74	0.391	0.005	[0.000, 0.040]
Fairness/cheating	3.76 (1.36)	3.83 (1.29)	0.13	0.723	0.001	[0.000, 0.023]
Loyalty/betrayal	1.84 (0.94)	2.13 (1.19)	2.76	0.099	0.018	[0.000, 0.068]
Authority/subversion	4.83 (1.33)	5.24 (1.20)	4.06	0.046	0.027	[0.0003, 0.082]
Sanctity/degradation	3.26 (1.41)	3.75 (1.59)	4.03	0.047	0.026	[0.0002, 0.082]
Overall punitiveness	3.53 (0.79)	3.82 (0.90)	4.35	0.039	0.028	[0.001, 0.085]

#### Mediation analyses

Expressed as point-biserial correlation coefficient, the nostalgia manipulation significantly increased moral concern, *r*(151) = 0.219, *p* = 0.007, 95% CI [0.061, 0.366] and punitiveness, *r*(151) = 0.168, *p* = 0.039, 95% CI [0.009, 0.320], when averaged across foundations. Furthermore, overall levels of moral concern and punitiveness were significantly correlated, *r*(151) = 0.195, *p* = 0.016, 95% CI [0.036, 0.344]. As a next step, we tested the indirect effect of nostalgia (vs. control) on overall punitiveness via overall moral concern, using the PROCESS macro (Model 4, 10,000 bootstrap samples). We contrast-coded the nostalgia manipulation (-1 = *control*, 1 = *nostalgia*). When controlling for nostalgia, the association between moral concern and punitiveness was significant (path *b*), b* = 0.166, *t*(148) = 2.03, *p* = 0.044, 95% CI [0.004, 0.328]. When controlling for moral concern, the direct effect of nostalgia was not significant (path *c’*), b* = 0.132,* t*(148) = 1.61, *p* = 0.109, 95% CI [-0.030, 0.293]. Consistent with H3, there was a significant indirect effect of nostalgia on punitiveness through moral concern (path *ab*), b* = 0.036, 95% CI = [0.001, 0.089].

#### Model comparison

To evaluate model fit, we tested two full-mediation models. The hypothesized model (nostalgia ⇒ moral concern ⇒ punitiveness) had excellent fit, Χ^2^(1) = 2.61, *p* = 0.106, SRMR = 0.051, AIC = 12.61, BIC = 27.70. Fit for the alternative model (nostalgia ⇒ punitiveness ⇒ moral concern) was worse than for the hypothesized model, Χ^2^(1) = 5.65, *p* = 0.017, SRMR = 0.076, AIC = 15.65, BIC = 30.74.

#### Ancillary content analysis

Participants provided detailed written descriptions of their nostalgic or ordinary experiences. This created an opportunity to corroborate the basic tenet that nostalgia prompts thoughts and concerns about others. Specifically, we content analyzed participants’ written narratives using the Linguistic Inquiry and Word Count software program^93^. Results indicated that references to sociality (e.g., “parent,” “friend”), as indexed by the program’s social referents category, were indeed more frequent in the nostalgia (*M* = 7.65, *SD* = 4.30) than control (*M* = 4.92, *SD* = 4.42) condition, *F*(1, 149) = 14.70, *p* < 0.001, η^2^ = 0.090, 90% CI [0.030, 0.168]. (Scores indicate the frequency of words in the social referents category as a percentage of total word count.)

### Discussion

Addressing the inherent limitations of cross-sectional Studies 1–2, Study 3 used an experimental design to determine nostalgia’s causal impact on moral concern and punitiveness, and to corroborate the emotion’s initiatory role within the hypothesized causal chain. Nostalgia (vs. control) increased both moral concern (H1) and punitiveness (H2), irrespective of moral foundation. As before, the link between nostalgia and punitiveness was numerically small but replicable. Crucially, as in Studies 1–2, moral concern mediated the effect of nostalgia (vs. control) on punitiveness (H3). Model comparisons again favored the hypothesized model over an alternative one that reversed the order of moral concern and punitiveness. In Study 4, we examined the generalizability of these findings.

## Study 4

Across Studies 1–3, we used the same measures to assess moral concern and punitiveness, respectively. This approach is beneficial from the viewpoint of assessing reproducibility and consistency within a set of studies. A drawback, however, is that it risks introducing mono-operation bias, thereby limiting generalizability^94^. To address this limitation, in Study 4, we used alternative operationalizations of moral concern (i.e., justice sensitivity) and punitiveness (i.e., attitudes toward criminal punishment). To further bolster generalizability, we relied on a representative sample of the Dutch public.

### Method

#### Participants

Participants were 341 Dutch participants (186 women, 155 men), who were enrolled in the Longitudinal Internet Studies for the Social Sciences (LISS) panel (www.lissdata.nl). It comprises household members selected based on a true probability sampling of households registered with Statistics Netherlands. Panel members complete studies each month, and their responses can be merged across studies. Data collection was managed by CentERdata in Tilburg, The Netherlands. The sample was heterogeneous with respect to age (range: 15–90 years, *M* = 50.31, *SD* = 17.37), relationship status (57% married, 27% single, 10% divorced, 6% widowed), and educational background (11% completed primary education only, 28% completed secondary education having received basic vocational training, 11% completed secondary education having received advanced vocational training, 50% completed college or university). We had no control over sample size, as it was determined by the number of panel members who completed the three scales of interest (specified below). Nonetheless, a sensitivity power analysis indicated that a sample size of 341 afforded 80% power to detect a small-to-medium effect size, *r* = 0.15 (two-tailed, α = 0.05; G*Power 3.1^95^).

#### Materials and procedure

We assembled the dataset from two LISS studies. Below, we report all measures we used for hypothesis testing. We derived measures of nostalgia and moral concern from the “Interpersonal Effects of Crying Part 2” study (administered May 2010). We derived the measure of punitiveness from the “Public Attitudes Towards and Knowledge of Conditional Sentences” study (administered October 2010). Demographic information is updated monthly in LISS, and we derived such information from the “Background Variables” study of May 2010.

**Nostalgia.** We assessed this construct with the SNS^61^. We averaged the seven items to form a nostalgia index (α = 0.95, *M* = 3.86, *SD* = 1.32).

**Moral Concern.** We assessed this construct with the 10-item Observer version of the Justice Sensitivity Scale^96^. Participants rated how angry they would be in various scenarios where others were being treated unfairly or put in a disadvantageous position (e.g., “I get very angry when someone is treated worse than somebody else;” 1 = *completely disagree*, 9 = *completely agree*). We reasoned that anger about injustice reflects the importance that one ascribes to being a good (vs. bad) person (i.e., moral concern). We averaged responses to create a moral concern index (α = 0.90, *M* = 6.29, *SD* = 1.25).

**Punitiveness.** We assessed this construct with the 13-item Punitiveness subscale of de Keijser’s Penal Attitudes Scale^97^. Participants indicated their agreement with several statements favoring criminal punishment (e.g., “An imposed sentence must really feel like punishment by the offender”; 1 = *completely disagree*, 5 = *completely agree*). We reasoned that higher scores on this scale reflect the importance of meting out severe punishment to a moral transgressor (i.e., punitiveness). We averaged responses to form a punitiveness index (α = 0.91, *M* = 4.23, *SD* = 0.64).

### Results

Consistent with H1, trait nostalgia was positively associated with moral concern, *r*(341) = 0.216, *p* < 0.001, 95% CI [0.112, 0.315]. Consistent with H2, trait nostalgia was positively related to punitiveness, *r*(341) = 0.144, *p* = 0.008, 95% CI [0.038, 0.246]. Moral concern, in turn, was positively linked with punitiveness, *r*(341) = 0.276, *p* < 0.001, 95% CI [0.175, 0.371], setting the stage for mediation analyses to test H3.

#### Mediation analyses

We used the PROCESS macro to test H3, namely, that moral concern mediates the link between trait nostalgia and punitiveness (Model 4, 10,000 bootstrap samples). When controlling for nostalgia, the association between moral concern and punitiveness was significant (path *b*), b* = 0.257, *t*(338) = 4.82, *p* < 0.001, 95% CI [0.152, 0.362]. When controlling for moral concern, the direct effect of trait nostalgia on punitiveness was not significant, (path *c’*), b* = 0.088,* t*(338) = 1.66, *p* = 0.099, 95% CI [-0.017, 0.193]. There was a significant indirect effect of nostalgia on punitiveness via moral concern (path *ab*), b* = 0.055, 95% CI = [0.020, 0.100]. The results are in accord with H3.

#### Model comparisons

Fit indices for the hypothesized model (nostalgia ⇒ moral concern ⇒ punitiveness) were excellent, Χ^2^(1) = 2.75, *p* = 0.097, SRMR = 0.034, AIC = 12.75, BIC = 31.91, and better than for the alternative model (nostalgia ⇒ punitiveness ⇒ moral concern), Χ^2^(1) = 11.82, *p* < 0.001, SRMR = 0.072, AIC = 21.82, BIC = 40.98.

### Discussion

By implementing alternative operationalizations of moral concern and punitiveness, Study 4 provided vital support for the generalizability of our theoretical framework beyond the moral-foundations based measures of Studies 1–3. In a diverse, nationally representative sample, nostalgia was positively associated with justice sensitivity (an index of moral concern), which, in turn, predicted harsher penal attitudes (an index of punitiveness).

### General discussion

Reactions to moral violations are influenced by several sources, including emotions. We focused on nostalgia. This emotion is imbued with sociality and begets sociality^21,22^. Relatedly, moral violations may come in several forms or foundations (Care/Harm, Fairness/Cheating, Loyalty/Betrayal, Authority/Subversion, and Sanctity/Degradation^31^), but all of them entail—to a varying degree—an assault on sociality, social norms, or the social order. We thus wondered how nostalgic individuals would respond to the threat on sociality and the social order inflicted by moral violations.

We offered three hypotheses, focused on establishing the relations among nostalgia, moral concern, and punitiveness toward moral transgressions. Specifically, nostalgia is linked to, or intensifies, both moral concern (H1) and punitiveness toward moral transgressions (H2); and the link between nostalgia and punitiveness toward moral transgressions is mediated by moral concern (H3). We obtained support for all hypotheses using multiple operationalizations of the relevant constructs (i.e., nostalgia, moral concern, punitiveness) and diverse samples (i.e., Prolific Academic workers from the U.K., U.S., and Canada, French business school students, representative Dutch sample). In Study 1, trait nostalgia was positively associated with moral concern and punishment intentions. Study 1 offered initial evidence, albeit correlational, for the notion that nostalgia intensifies moral concern (H1) and ensuing punitiveness (H2). Model comparisons favored the hypothesized model in which the effect of nostalgia on punitiveness is mediated by moral concern (H3), over a model in which the order of moral concern and punitiveness was reversed. Preregistered Study 2 replicated these preliminary findings and extended them by using three convergent measures of trait nostalgia, bolstering construct validity. On the basis of an experimental design, we showed in Study 3 that participants who had reflected on a nostalgic (compared to ordinary) autobiographical event reported higher levels of moral concern and punitiveness. Nostalgia’s effect on increased punitiveness was transmitted by intensified moral concern. The experimental evidence corroborated nostalgia’s initiatory role in the hypothesized causal chain. Based on secondary data from a representative Dutch sample, Study 4 more broadly tested and supported our theoretical framework with alternative operationalizations of moral concern (as justice sensitivity) and punitiveness (as penal attitudes).

The findings enrich the morality literature, as they point to another (besides anger, contempt, disgust, gratitude, shame, guilt, or embarrassment^5–7,10,57^) discrete emotion, that of nostalgia, as a precursor of moral judgments and punitiveness. The findings also enrich the nostalgia literature, as they demonstrate that nostalgia protects social relationships by endorsing punishment for those who commit moral violations. Nostalgia serves, in part, to sustain social bonds and norms. When these are threatened by moral violations, nostalgia renders the punishment harsher.

Future work will need to test the generalizability and boundaries of our findings. First, across studies, the direct effect of nostalgia on punitiveness was small and became non-significant when moral concern was included in the model (i.e., was mediated by moral concern, as hypothesized). Nostalgia, then, is only weakly related to punitiveness. We argued that nostalgia, due to its approach- and future-oriented property, allows people to stand up and defend their moral values in part through punitiveness. Had we offered participants an alternative and prosocial choice, such as forgiveness, they might have taken it. Future research should consider providing participants with both punitiveness and forgiveness options. Second, participants evaluated generalized scenarios in which we did not specify that the transgressor belonged to an ingroup or an outgroup. It is possible that, during nostalgic reverie, individuals are more likely to be forgiving toward ingroup than outgroup members, or be more punitive toward outgroup than ingroup members^65^. Third, we did not distinguish between different types of punishment (e.g., utilitarian, retributive, deontological, or instrumental^70^). Follow-up research will do well to explore the generalizability of nostalgia across types of punishment. Fourth, we established that nostalgia is associated with, or increases, moral concern and punitiveness toward moral transgressions. Future investigations could test whether nostalgia prioritizes adherence to prescriptive norms, including those against moral violations. Fifth, our work demonstrated a foundation-general effect of nostalgia on moral concern and punitiveness. Future research could examine circumstances under which nostalgia may have domain-specific consequences. Finally, and related to the previous point, future research could consider a sixth moral foundation, Liberty, which emphasizes personal freedom and the right to make autonomous decisions without undue interference^98^. Nostalgia, which is associated with interdependent self-construal^25^, may be antithetical to the Liberty foundation, which emphasizes independence and self-reliance. If so, a domain-specific (null) effect is plausible.

In conclusion, nostalgia, a frequently experienced and social emotion that spans across ages and cultures, serves partly to keep one’s moral compass in check. Nostalgia intensifies moral concern and, by so doing, increases punitiveness toward moral transgressors.

### Supplementary Information

Supplementary Information.

## Data Availability

All stimulus materials, data and analysis codes for the reported studies are available on Open Science Framework (https://osf.io/nujah/?view_only=2f65a757b8b34555a95d127bf16b3e13).

## References

[1] Donner M, Chagas-Bastos FH, Jeremiah R, Laham S (2023). Pathogens or promiscuity? Testing two accounts of the relation between disgust sensitivity and binding moral values. Emotion.

[2] Horberg EJ, Oveis C, Keltner D, Cohen AB (2009). Disgust and the moralization of purity. J. Pers. Soc. Psychol..

[3] Landy JF, Goodwin GP (2015). Does incidental disgust amplify moral judgment? A meta-analytic review of experimental evidence. Perspect. Psychol. Sci..

[4] Molho C, Tybur JM, Güler E, Balliet D, Hofmann W (2017). Disgust and anger relate to different aggressive responses to moral violations. Psychol. Sci..

[5] Rozin P, Lowery L, Imada S, Haidt J (1999). The CAD triad hypothesis: A mapping between three moral emotions (contempt, anger, disgust) and three moral codes (community, autonomy, divinity). J. Pers. Soc. Psychol..

[6] Russell PS, Giner-Sorolla R (2013). Bodily moral disgust: What it is, how it is different from anger, and why it is an unreasoned emotion. Psychol. Bull..

[7] Jackson L (2016). Why should I be grateful? The morality of gratitude in contexts marked by injustice. J. Moral Educ..

[8] McCullough ME, Kilpatrick SD, Emmons RA, Larson DB (2001). Is gratitude a moral affect?. Psychol. Bull..

[9] Malti T, Krettenauer T (2013). The relation of moral emotion attributions to prosocial and antisocial behavior: A meta-analysis. Child Dev..

[10] Tangney JP, Stuewig J, Mashek DJ (2007). Moral emotions and moral behavior. Annu. Rev. Psychol..

[11] Pearsall J, Hanks P (1998). The New Oxford Dictionary of English.

[12] Hepper EG, Ritchie TD, Sedikides C, Wildschut T (2012). Odyssey’s end: Lay conceptions of nostalgia reflect its original Homeric meaning. Emotion.

[13] Wildschut T, Sedikides C, Arndt J, Routledge C (2006). Nostalgia: Content, triggers, functions. J. Pers. Soc. Psychol..

[14] Hepper EG (2014). Pancultural nostalgia: Prototypical conceptions across cultures. Emotion.

[15] Sedikides C, Wildschut T (2022). Nostalgia across cultures. J. Pac. Rim Psychol..

[16] Madoglou A, Gkinopoulos T, Xanthopoulos P, Kalamaras D (2017). Representations of autobiographical nostalgic memories: Generational effect, gender, nostalgia proneness and communication of nostalgic experiences. J. Integr. Soc. Sci..

[17] Turner JR, Stanley JT (2021). Holding on to pieces of the past: Daily reports of nostalgia in a life-span sample. Emotion.

[18] Kaplan HA (1987). The psychopathology of nostalgia. Psychoanal. Rev..

[19] Batcho KI (1998). Personal nostalgia, world view, memory, and emotionality. Percept. Mot. Skills.

[20] Leunissen J, Wildschut T, Sedikides C, Routledge C (2021). The hedonic character of nostalgia: An integrative data analysis. Emot. Rev..

[21] Juhl J, Biskas M (2023). Nostalgia: An impactful social emotion. Curr. Opin. Psychol..

[22] Sedikides C, Wildschut T (2019). The sociality of personal and collective nostalgia. Eur. Rev. Soc. Psychol..

[23] Lasaleta JD, Sedikides C, Vohs KD (2014). Nostalgia weakens the desire for money. J. Consum. Res..

[24] Wildschut T, Sedikides C, Kelley NJ (2023). Trait nostalgia: Four scales and a recommendation. Curr. Opin. Psychol..

[25] Abakoumkin G, Wildschut T, Sedikides C (2020). Nostalgia proneness and the collective self. Front. Psychol..

[26] Sedikides C, Brewer MB (2015). Individual Self, Relational Self, Collective Self.

[27] Hertz DG (1990). Trauma and nostalgia: New aspects on the coping of aging Holocaust survivors. Isr. J. Psychiatry Relat. Sci..

[28] Sedikides C, Wildschut T, Baden D (2004). Nostalgia: Conceptual Issues and Existential Functions. Handbook of Experimental Existential Psychology.

[29] Cheung W-Y, Wildschut T, Sedikides C (2018). Autobiographical memory functions of nostalgia in comparison to rumination and counterfactual thinking: Similarity and uniqueness. Memory.

[30] Van Dijke M, Wildschut T, Leunissen JM, Sedikides C (2015). Nostalgia buffers the negative impact of low procedural justice on cooperation. Organ. Behav. Hum. Decis. Process..

[31] Graham J (2013). Moral Foundations Theory: The Pragmatic Validity of Moral Pluralism. Advances in Experimental Social Psychology.

[32] Haidt J, Graham J, Joseph C (2009). Above and below left–right: Ideological narratives and moral foundations. Psychol. Inq..

[33] Curry OS, Mullins DA, Whitehouse H (2019). Is it good to cooperate? Testing the theory of morality-as-cooperation in 60 societies. Curr. Anthropol..

[34] Ellemers N (2017). Morality and the Regulation of Social Behavior: Groups as Moral Anchors.

[35] Tomasello M, Vaish A (2013). Origins of human cooperation and morality. Annu. Rev. Psychol..

[36] Day MV, Fiske ST, Downing EL, Trail TE (2014). Shifting liberal and conservative attitudes using moral foundations theory. Pers. Soc. Psychol. Bull..

[37] Winterich KP, Aquino K, Mittal V, Swartz R (2013). When moral identity symbolization motivates prosocial behavior: The role of recognition and moral identity internalization. J. Appl. Psychol..

[38] Steffens J (2020). The influence of film music on moral judgments of movie scenes and felt emotions. Psychol. Music.

[39] Doğruyol B, Alper S, Yilmaz O (2019). The five-factor model of the moral foundations theory is stable across WEIRD and non-WEIRD cultures. Personal. Individ. Differ..

[40] Aquino K, Reed A (2002). The self-importance of moral identity. J. Pers. Soc. Psychol..

[41] Kohlberg, L. From is to out: How to commit the naturalistic fallacy and get away with it in the study of moral development. *Cogn. Dev. Epistemol.* (1971).

[42] Suhler CL, Churchland P (2011). Can innate, modular “foundations” explain morality? Challenges for Haidt’s moral foundations theory. J. Cogn. Neurosci..

[43] Gray K, Waytz A, Young L (2012). The moral dyad: A fundamental template unifying moral judgment. Psychol. Inq..

[44] Davis DE (2016). The moral foundations hypothesis does not replicate well in Black samples. J. Pers. Soc. Psychol..

[45] Davies CL, Sibley CG, Liu JH (2014). Confirmatory factor analysis of the moral foundations questionnaire. Soc. Psychol..

[46] Iurino K, Saucier G (2020). Testing measurement invariance of the moral foundations questionnaire across 27 countries. Assessment.

[47] Yilmaz O, Harma M, Bahçekapili HG, Cesur S (2016). Validation of the moral foundations questionnaire in Turkey and its relation to cultural schemas of individualism and collectivism. Pers. Individ. Differ..

[48] Yudkin DA, Gantman AP, Hofmann W, Quoidbach J (2021). Binding moral values gain importance in the presence of close others. Nat. Commun..

[49] Abeyta AA, Routledge C, Juhl J (2015). Looking back to move forward: Nostalgia as a psychological resource for promoting relationship goals and overcoming relationship challenges. J. Pers. Soc. Psychol..

[50] Leunissen JM, Van Dijke M, Wildschut T, Sedikides C (2023). Organisational nostalgia: The construct, the scale, and its implications for organizational functioning. Br. J. Manag..

[51] Juhl J (2020). Nostalgia proneness and empathy: Generality, underlying mechanism, and implications for prosocial behavior. J. Pers..

[52] Zhou X, Wildschut T, Sedikides C, Shi K, Feng C (2012). Nostalgia: The gift that keeps on giving. J. Consum. Res..

[53] Cheung W-Y, Sedikides C, Wildschut T, Tausch N, Ayanian AH (2017). Collective nostalgia is associated with stronger outgroup-directed anger and participation in ingroup-favoring collective action. J. Soc. Polit. Psychol..

[54] Turner R, Wildschut T, Sedikides C (2022). Reducing social distance caused by weight stigma: Nostalgia changes behavior toward overweight individuals. J. Appl. Soc. Psychol..

[55] Turner RN, Wildschut T, Sedikides C, Gheorghiu M (2013). Combating the mental health stigma with nostalgia. Eur. J. Soc. Psychol..

[56] Turner RN, Wildschut T, Sedikides C (2018). Fighting ageism through nostalgia. Eur. J. Soc. Psychol..

[57] Horberg EJ, Oveis C, Keltner D (2011). Emotions as moral amplifiers: An appraisal tendency approach to the influences of distinct emotions upon moral judgment. Emot. Rev..

[58] Batson CD, Ahmad N, Lishner DA (2009). Empathy and Altruism. Oxford Handbook of Positive Psychology.

[59] Frijda NH, Mesquita B (1998). The Analysis of Emotions: Dimensions of Variation. What Develops in Emotional Development?.

[60] Haidt J (2003). Elevation and the Positive Psychology of Morality. Flourishing: Positive Psychology and the Life Well-Lived.

[61] Sedikides C (2015). To Nostalgize: Mixing Memory with Affect and Desire. Advances in Experimental Social Psychology.

[62] Turner RN, Stathi S (2023). Nostalgic intergroup contact and intergroup relations: theoretical, empirical, and applied dimensions. Curr. Opin. Psychol..

[63] Martinovic B, Jetten J, Smeekes A, Verkuyten M (2017). Collective memory of a dissolved country: Group-based nostalgia and guilt assignment as predictors of interethnic relations between diaspora groups from former Yugoslavia. J. Soc. Polit. Psychol..

[64] Smeekes A, Sedikides C, Wildschut T (2023). Collective nostalgia: Triggers and consequences for collective action intentions. Br. J. Soc. Psychol..

[65] Wildschut T, Bruder M, Robertson S, van Tilburg WA, Sedikides C (2014). Collective nostalgia: A group-level emotion that confers unique benefits on the group. J. Pers. Soc. Psychol..

[66] Leunissen JM, Sedikides C, Wildschut T, Cohen TR (2018). Organizational nostalgia lowers turnover intentions by increasing work meaning: The moderating role of burnout. J. Occup. Health Psychol..

[67] Abeyta AA, Pillarisetty S (2023). Nostalgia supports a meaningful life. Curr. Opin. Psychol..

[68] Sedikides C, Wildschut T (2018). Finding meaning in nostalgia. Rev. Gen. Psychol..

[69] Yin Y, Jiang T, Thomaes S, Wildschut T, Sedikides C (2023). Nostalgia promotes parents’ tradition transfer to children: The role of self-child overlap. Pers. Soc. Psychol. Bull..

[70] Giacomantonio M, Pierro A, Baldner C, Kruglanski A (2017). Need for closure, torture, and punishment motivations: The mediating role of moral foundations. Soc. Psychol..

[71] Haidt J, Graham J (2007). When morality opposes justice: Conservatives have moral intuitions that liberals may not recognize. Soc. Justice Res..

[72] Koleva SP, Graham J, Iyer R, Ditto PH, Haidt J (2012). Tracing the threads: How five moral concerns (especially Purity) help explain culture war attitudes. J. Res. Pers..

[73] Konishi N, Oe T, Shimizu H, Tanaka K, Ohtsubo Y (2017). Perceived shared condemnation intensifies punitive moral emotions. Sci. Rep..

[74] Batcho KI (2023). Nostalgia in literature and memoir. Curr. Opin. Psychol..

[75] Sedikides C, Wildschut T (2016). Past forward: Nostalgia as a motivational force. Trends Cogn. Sci..

[76] Sedikides C, Wildschut T (2020). The motivational potency of nostalgia: The future is called yesterday. Advances in motivation science.

[77] Kazak AE (2018). Journal article reporting standards. Am. Psychol..

[78] Schönbrodt FD, Perugini M (2013). At what sample size do correlations stabilize?. J. Res. Personal..

[79] Kenny, D. A. An interactive tool for the estimation of power in tests of mediation. (2017).

[80] Graham J, Haidt J, Nosek BA (2009). Liberals and conservatives rely on different sets of moral foundations. J. Pers. Soc. Psychol..

[81] Steiger JH (2004). Beyond the F test: Effect size confidence intervals and tests of close fit in the analysis of variance and contrast analysis. Psychol. Methods.

[82] Hayes AF (2022). Introduction to Mediation, Moderation, and Conditional Process Analysis: A Regression-Based Approach.

[83] Rohrer JM, Hünermund P, Arslan RC, Elson M (2022). That’s a lot to process! Pitfalls of popular path models. Adv. Methods Pract. Psychol. Sci..

[84] Kenny DA, Kaniskan B, McCoach DB (2015). The performance of RMSEA in models with small degrees of freedom. Sociol. Methods Res..

[85] Hu L, Bentler PM (1999). Cutoff criteria for fit indexes in covariance structure analysis: Conventional criteria versus new alternatives. Struct. Equ. Model. Multidiscip. J..

[86] Akaike H (1974). A new look at the statistical model identification. IEEE Trans. Autom. Control.

[87] Schwarz G (1978). Estimating the dimension of a model. Ann. Stat..

[88] Campbell DT, Fiske DW (1959). Convergent and discriminant validation by the multitrait-multimethod matrix. Psychol. Bull..

[89] Wildschut T, Sedikides C (2022). Measuring Nostalgia. Handbook of Positive Psychology Assessment.

[90] Kelley NJ (2022). Nostalgia confers psychological wellbeing by increasing authenticity. J. Exp. Soc. Psychol..

[91] Newman DB, Sachs ME, Stone AA, Schwarz N (2020). Nostalgia and well-being in daily life: An ecological validity perspective. J. Pers. Soc. Psychol..

[92] Mehrabian A, Epstein N (1972). A measure of emotional empathy. J. Pers..

[93] Pennebaker, J. W., Booth, R. J. & Francis, M. E. Linguistic inquiry and word count: LIWC [Computer software]. *Austin TX Liwc Net* **135**, (2007).

[94] Shadish, W. R., Cook, T. D. & Campbell, D. T. *Experimental and Quasi-Experimental Designs for Generalized Causal Inference*. xxi, 623 (Houghton, Mifflin and Company, Boston, MA, US, 2002).

[95] Faul F, Erdfelder E, Lang A-G, Buchner A (2007). G*Power 3: A flexible statistical power analysis program for the social, behavioral, and biomedical sciences. Behav. Res. Methods.

[96] Schmitt M, Gollwitzer M, Maes J, Arbach D (2005). Justice sensitivity. Eur. J. Psychol. Assess..

[97] Van Gelder J-L, Aarten P, Lamet W, Van Der Laan P (2015). Unknown, unloved? Public opinion on and knowledge of suspended sentences in the Netherlands. Crime Delinquency.

[98] Haidt J (2012). The Righteous Mind: Why Good People Are Divided by Politics and Religion.

