# Supplementary material for: Nostalgia increases punitiveness by intensifying moral concern
Source: Sci Rep. 2024 May 19;14:11425. doi: 10.1038/s41598-024-61858-x (PMC11102900; doi:10.1038/s41598-024-61858-x)
Supplement: Supplementary file 1 — Supplementary Information. [file 41598_2024_61858_MOESM1_ESM.docx]

**SUPPLEMENTARY INFORMATION**

**Nostalgia Increases Punitiveness by Intensifying Moral Concern**

**Table S1**

*Associations of Three Nostalgia Scales with Moral Concern in Study 2*

|  | SNS | |  | PINE | |  | NPS | |
| --- | --- | --- | --- | --- | --- | --- | --- | --- |
|  | *r* | 95% CI |  | *r* | 95% CI |  | *r* | 95% CI |
| Care/Harm | .104^+^ | [-.018, .223] |  | .135^*^ | [.013, .253] |  | .222^***^ | [.102, .335] |
| Fairness/Cheating | .055 | [-.067, .176] |  | .055 | [-.068, .176] |  | .089 | [-.034, .209] |
| Loyalty/Betrayal | .254^***^ | [.136, .365] |  | .315^***^ | [.201, .421] |  | .326^***^ | [.213, .431] |
| Authority/Subversion | .178^**^ | [.057, .294] |  | .256^***^ | [.139, .367] |  | .281^***^ | [.165, .390] |
| Sanctity/Degradation | .226^***^ | [.107, .339] |  | .273^***^ | [.156, .382] |  | .310^***^ | [.195, .416] |
| Overall moral concern | .255^***^ | [.137, .366] |  | .322^***^ | [.208, .428] |  | .377^***^ | [.268, .477] |

*Note.* SNS = Southampton Nostalgia Scale. PINE = Personal Inventory of Nostalgic Experiences. NPS = Nostalgia Prototype Scale.

^+^ *p* < .10. * *p* < .05. ** *p* < .01. *** *p* < .001.

**Table S2**

*Associations of Three Nostalgia Scales with Punitiveness in Study 2*

|  | SNS | |  | PINE | |  | NPS | |
| --- | --- | --- | --- | --- | --- | --- | --- | --- |
|  | *r* | 95% CI |  | *r* | 95% CI |  | *r* | 95% CI |
| Care/Harm | .057 | -.065, .178 |  | .085 | -.037, .205 |  | .092 | -.030, .212 |
| Fairness/Cheating | .032 | -.091, .153 |  | .006 | -.116, .128 |  | .039 | -.084, .160 |
| Loyalty/Betrayal | .137^*^ | .015, .255 |  | .171^**^ | .050, .288 |  | .180^**^ | .059, .295 |
| Authority/Subversion | .053 | -.070, .174 |  | .064 | -.058, .185 |  | .085 | -.038, .205 |
| Sanctity/Degradation | .129^*^ | .007, .247 |  | .180^**^ | .059, .296 |  | .225^***^ | .106, .338 |
| Overall moral concern | .113^+^ | -.009, .232 |  | .142^*^ | .020, .260 |  | .179^**^ | .058, .295 |

*Note.* SNS = Southampton Nostalgia Scale. PINE = Personal Inventory of Nostalgic Experiences. NPS = Nostalgia Prototype Scale.

^+^ *p* < .10. * *p* < .05. ** *p* < .01. *** *p* < .001.

**Table S3**

*Indirect Effects of Three Nostalgia Scales on Overall Punitiveness via Overall Moral Concern in Study 2*

| Nostalgia scale | Indirect effect: *ab* | bootstrap 95% CI |
| --- | --- | --- |
| SNS | .107 | [.049, .176] |
| PINE | .135 | [.071, .211] |
| NPS | .155 | [.090, .232] |

*Note.* SNS = Southampton Nostalgia Scale. PINE = Personal Inventory of Nostalgic Experiences. NPS = Nostalgia Prototype Scale. Indirect effects are statistically significant when 95% CI excludes 0.
